# A History of Trauma is Associated with Aggression, Depression, Non-Suicidal Self-Injury Behavior, and Suicide Ideation in First-Episode Psychosis

**DOI:** 10.3390/jcm8071082

**Published:** 2019-07-23

**Authors:** Rebecca E. Grattan, Natalia Lara, Renata M. Botello, Valerie L. Tryon, Adrienne M. Maguire, Cameron S. Carter, Tara A. Niendam

**Affiliations:** Department of Psychiatry & Behavioral Sciences, University of California-Davis, Sacramento, CA 95817, USA

**Keywords:** trauma, depression, aggression, suicide ideation, suicide behavior, non-suicidal self-injury behavior, first-episode psychosis

## Abstract

The association between trauma and psychosis outcomes is well-established, and yet the impact of trauma on comorbid clinical symptoms—such as aggression, non-suicidal self-injury behavior (NSSIB), suicide ideation, and suicide behavior—for those with psychosis is unclear. To effectively treat those with first-episode psychosis (FEP) and a history of trauma, we need to understand the impact of trauma on their whole presentation. FEP participants were recruited from an Early Psychosis Program (*N* = 187, ages 12–35, 72.2% male). Clinicians gathered history of trauma, aggression, and suicide data, and rated current symptom severity and functioning. Data was coded using clinician rated measures, self-report measures, and retrospective clinical chart review. Regression analyses examined whether trauma was associated with a history of aggression, suicidal ideation, suicide behavior, NSSIB, symptoms, and functioning. Trauma was associated with aggression, aggression severity and type of aggression (aggression towards others). Trauma was also associated with depression severity, suicide ideation, most severe suicide ideation, and NSSIB. Trauma was not associated with suicide behavior, severity of suicide behavior or psychosocial functioning. Integrating trauma treatment into FEP care could reduce rates of depression, aggression, suicide ideation, and NSSIB for those with a history of trauma. To reduce suicide attempt occurrence and improve functioning, more research is needed.

## 1. Introduction

The association between trauma and psychosis outcomes is well-established. Traumatic life events are linked with increased odds of developing a psychotic disorder [1], and individuals with psychotic disorders are significantly more likely to report a history of trauma [2]. Rates of post-traumatic stress disorder (PTSD) in people with schizophrenia are elevated compared to the general population [3], and traumatic life events are also associated with higher rates of psychosis symptoms [2].

Despite the wealth of research linking trauma and subsequent development of psychosis, the impact of trauma on the initial clinical presentation of psychosis is less clear. At initial contact for treatment, a history of trauma may be associated with a different pattern of challenging symptoms or behaviors that may require different treatment approaches. Given these challenging behaviors can interfere with therapy and everyday functioning [4], the impact of trauma on the clinical presentation of first-episode psychosis (FEP) is vital to guide decision-making for treatment selection and process.

### 1.1. Impact of Trauma on Non-Psychosis Populations

Studies in both general and psychiatric populations show that those who have experienced trauma show higher rates of challenging symptoms and behaviors. Traumatic life events are associated with self-harming behaviors [5,6], aggression [7], increased substance use [8], risky sexual behavior [9], suicide behavior [10], suicide ideation [11], homelessness [12], and impulsivity [7].

There is mixed evidence regarding the impact of trauma on psychosocial functioning. While some studies, such as studies of war refugees, find no relationship between the number of traumatic events and social functioning scores [13], many studies support a relationship between psychosocial functioning and traumatic life event exposure. In individuals with severe mental illness [12] or personality disorders [14], negative life events are associated with poorer psychosocial functioning in general and over time. The impact of trauma on psychosocial functioning is dependent on prior psychosocial functioning and the mental health of the individual [15], which may explain some of the discrepancies in the literature. Additionally, higher levels of PTSD symptoms are associated with less perceived support from friends in a veteran population, which could provide a possible explanation for reports of declining social functioning [16].

### 1.2. Impact of Trauma on Psychosis Populations

Few studies have examined clinical correlates of trauma in the early stages of psychotic illness. Suicide behavior and ideation are associated with a history of trauma in individuals with schizophrenia when controlling for depression [17]. Similarly, a history of trauma in those at Clinical High Risk (CHR) for psychosis is associated with increased suicide ideation, but not suicide behavior [18], although it is unclear whether other variables such as depression could account for this relationship. In both Grivel et al. [18] and Mohammadzadeh et al. [17], other related variables such as non-suicidal self-injury behavior (NSSIB) are not measured. In FEP populations, trauma history is associated with both past [19] and future suicide attempts [20], but the role of trauma on suicide ideation remains unclear. To our knowledge, the relationship between trauma and other suicide variables or NSSIB has not been examined in FEP populations.

In terms of aggression, in veteran populations, those with comorbid psychosis and PTSD have higher self-reported feelings and actions of aggression than those with only PTSD or psychosis [21]. Similarly, in adults experiencing psychosis, sexual abuse, and community conflict are highly associated with aggression [22]. Additionally, in individuals with FEP, physical and sexual abuse is associated with a history of increased aggression [23]. Importantly, evidence shows that the most common type of aggression found in populations with psychosis is reactive aggression [24]. Reactive aggression refers to behaving aggressively in reaction to a perceived provocation or threat, in contrast to proactive aggression, which is behaving aggressively to achieve a particular goal [25].

Trauma is also associated with increases in comorbid symptoms, such as irritability [26], depression [27], anxiety [27,28], and general distress [29] in FEP populations. A possible explanatory mechanism for this relationship is increased stress sensitivity and emotion dysregulation. Those with higher stress sensitivity report more negative emotions in response to stressors, and trauma is associated with increased stress sensitivity [30].

In terms of functioning, there is little research examining the link between trauma and psychosocial functioning in people with FEP. In individuals with schizophrenia, PTSD symptoms are associated with various aspects of psychosocial functioning, including impaired patient-rated interpersonal and occupational functioning, but not interviewer-rated psychosocial functioning scores [3]. Victimization has also been found to be associated with poorer social and role functioning in men with schizophrenia [31]. Sexual abuse in particular seems to be linked with poorer role functioning and social functioning skills for adults with schizophrenia [32]. There is mixed evidence in FEP and CHR populations. In FEP, a history of trauma is associated with a decreased likelihood of working or living with family at entry to treatment, but it was not associated with improvements in functioning at discharge [33]. Similarly, in FEP, interpersonal childhood trauma is related to lower levels of family and social relationship satisfaction, and history of any childhood trauma is associated with poorer social functioning in early stages of development and poor academic functioning starting in early adolescence [34]. In CHR populations, one study found a relationship between traumatic life events and poor functioning [35], and another found no relationship [18].

The experience of trauma is linked with numerous biological and cognitive/belief-based changes, resulting in an altered developmental trajectory [36]. Building on the model proposed by Mayo et al. [36], biological changes, such as sensitization of the hypothalamic pituitary adrenal (HPA) axis and belief-based changes, such as altered schemas about ones safety in the world, can increase stress sensitivity and emotion dysregulation. The clinical correlates discussed above (e.g., NSSIB or aggression) could be conceptualized as poor coping strategies, developed to manage this dysregulation. Supporting this idea, suicide ideation and behavior [37], NSSIB [38], and aggression [39] are associated with emotion dysregulation. To reduce rates of suicide ideation and behavior, NSSIB, aggression and other negative emotions such as depression in first-episode psychosis populations, intervention may be most effective at the level of biological and belief changes, which may serve to address stress sensitization and emotion dysregulation.

### 1.3. Impact of Trauma on Treatment Engagement for Psychosis Populations

The impact of trauma on the treatment of psychosis is complex. In FEP, a history of physical or sexual trauma is linked to poorer treatment adherence [33]. Trauma history is also associated with reduced medication adherence and poor service engagement in those experiencing early psychosis [4]. Trauma history is also associated with poorer therapeutic alliance in individuals with schizophrenia [40]. However, data from a large cohort study of FEP individuals served by the Australian EPPIC program suggests that trauma history does not appear to impact role functioning after treatment in an early psychosis treatment program [33]. Further, there is no difference in level of childhood trauma in responders and non-responders to antipsychotic treatment [41]. Thus, understanding the impact of trauma on initial psychosis presentation and treatment engagement is vital if we seek to develop better methods to engage and treat individuals with psychosis who have been impacted by trauma.

### 1.4. Hypotheses

The aim of the present study is to characterize differences in common clinical correlates and functioning in an early psychosis group with and without a history of trauma, to support efforts to better understand, and therefore treat, this population. Specifically, we hypothesize that those who have experienced trauma will report higher historical rates of aggression, self-harm, suicide ideation, and suicide behavior at initial treatment presentation. We hypothesize that those with a trauma history will also report more severe non-psychotic symptoms, aggression, suicide ideation and behavior and poorer social and role functioning.

## 2. Methods

### 2.1. Participants

We recruited 798 individuals from the University of California, Davis, Early Diagnosis and Preventive Treatment (EDAPT) Clinic, an outpatient early psychosis clinic between October 2004 and December 2018. Participants were aged 12–35 and were assessed with the Structured Clinical Interview for DSM-IV-TR (SCID-IV) [42] or the Kiddie Schedule for Affective Disorders and Schizophrenia (K-SADS) [43] to determine eligibility as first-episode psychosis (FEP; onset of psychosis in the past 2 years) and received primary psychotic (schizophrenia, schizoaffective, schizophreniform disorders), other psychotic disorder not otherwise specified (NOS), or mood disorder with psychotic features (bipolar or major depression) diagnoses according to the DSM-IV-TR.

Participants were excluded if they were not comfortable speaking English, if they had a diagnosis of substance-induced psychosis, a neurological illness, any history of head trauma leading to unconsciousness, a Weschler Abbreviated Scale Intelligence (WASI) IQ below 70, or did not meet criteria for a FEP diagnosis (*N* = 283) [44]. If eligible after the clinical interview (*N* = 515), participants were invited to complete additional research appointments. Participants gave written informed consent, or assent in combination with parental consent (for those under 18), for their data to be collected using clinical measures and retrospective chart review, as part of a larger study of cognition in psychotic disorders. Participants for this study were selected if they had complete data for all variables of interest (*N* = 187). A subset (*N* = 107) of these participants were also included in Lopez-Garcia et al. [45]. This study was reviewed and approved by the UC Davis Institutional Review Board (protocol #226043).

### 2.2. Measures

Demographic information and psychiatric history were collected by clinicians (clinical psychologists, psychiatrists, and master’s level clinicians) during baseline clinical interview using the Structured Interview for Prodromal Symptoms (SIPS) [46] and SCID-IV [42]. Clinicians had demonstrated reliability on the SIPS and SCID-IV as defined by kappa of at least 0.70. For a subset of this population (*N* = 187), additional data were collected for the current research study. Trauma history was collected by clinicians at baseline using a combination of clinical interview, the SCID-IV and/or the PTSD checklist (PCL) [47]. Trauma was defined as exposure to, witnessing, or hearing of death, serious injury, or sexual violence as described in the DSM-TR-IV. Neglect, exposure to general violence, and trauma related to medical procedures was also coded. Aggression data were collected by clinicians at baseline using clinical interview. Suicide variables (suicide ideation and behavior) and non-suicidal self-harm behavior (NSSIB) data were collected by clinicians at baseline using the Columbia-Suicide Severity Rating Scale (C-SSRS) [48]. Clinicians were trained to reliability, defined as a kappa of at least 0.80, for rating agreement on the C-SSRS. Suicide data were coded for presence or absence of suicide ideation, behavior, and NSSIB as defined in the C-SSRS [48]. Most severe suicide ideation type reported (0 = No ideation; 1 = Wish to be dead; 2 = Non-specific active suicidal thoughts; 3 = Active suicide ideation with methods but no intent; 4 = Active suicidal ideation with some intent but no plan; 5 = Active suicidal ideation with specific plan and intent) and most severe suicide behavior type reported (0 = No behavior; 1 = Preparatory behavior; 2 = Aborted/Self-interrupted attempt; 3 = Interrupted attempt; 4 = Actual attempt) were also coded. Measures of symptoms and functioning were also captured at baseline using the Brief Psychiatric Rating Scale (BPRS) [49] and the Global Functioning: Social and Role scale [50], respectively. BPRS factor scores were used [51]; however, given the affect factor score includes an item on suicide, the depression BPRS item score was used instead of the affect factor score to avoid confounding relationships with suicide variables. Clinicians had demonstrated reliability on the BPRS as defined by an intraclass correlation coefficient of at least 0.7.

Trauma and aggression data were coded retrospectively, using a standardized chart review, based on all available information (validated measures, clinical interviews with the individual and collateral and other clinical and research data) similar to approaches described by Grivel et al. [18] and Keane et al. [52]. Research staff were trained on a practice data set to ensure consistency. Trauma history was coded as presence or absence of a trauma history as defined in the PTSD checklist [48]. Type of trauma (emotional, physical, sexual or other) was also recorded. For analyses, trauma history was considered as present or absent, as the power for group analyses by type of trauma was insufficient.

Aggression data were coded for presence or absence of aggression, using criteria from the Modified Overt Aggression Scale (MOAS) to determine presence of aggression [53]. Aggression type was also coded based on MOAS criteria, including physical and verbal aggression towards others, self, both self and others, and aggression towards objects. Severity of aggression was coded using a 10-point severity scale reported by Lopez-Garcia et al. [45] based on the MOAS. To compare the impact of aggression towards others as opposed to aggression towards self (suicide attempts and NSSIB), we also coded aggression severity that did not include suicide attempts or NSSIB. The following criteria were used to code for the most severe history of aggression within the domains described above.

1. No aggression.
2. Verbal aggression: yelling, screaming, cussing, argumentative.
3. Verbal aggression with threat: threatening harm towards self or others, but no weapon or action.
4. Verbal Aggression with threat/weapon: verbally threatening other with weapon, but no action.
5. Aggression against property: slamming doors, ripping clothing, throwing objects, breaking small objects, fire setting.
6. Aggression against property accompanied by threat: destroying large items with threat to do more.
7. Autoaggression: banging head, pounding walls, banging fists, pulling hair out.
8. Autoaggression (Self-harmarm with injury): punching wall (e.g., breaking hand), cutting/burning self (NSSIB), and aborted suicide attempts.
9. Physical aggression: pushing others, shaking others, hitting, kicking, scratching, and pinning down.
10. Physical aggression (with threat or injury): hitting and kicking people with threat to do more, suicide attempts; causing injury, potentially or actually lethal.

### 2.3. Statistical Analysis

Preliminary analyses examined data for outliers and determined if data met assumptions of normality, linearity, homoscedasticity, and multicollinearity as appropriate. Descriptive and inferential statistics (including chi-square analyses, ANOVA, Pearson correlations, and independent samples *t*-tests) were performed to determine demographic differences between groups (those with and without a history of trauma, and those with and without a history of clinical characteristics). Analyses examining relationships between demographic variables and clinical variables are presented in the Supplementary Materials. Hierarchical binomial logistic regressions examined relationships between trauma and categorical variables (suicide ideation, suicide behavior, NSSIB, and aggression). ANOVA examined differences in trauma history by aggression type. Ordinal logistic regressions examined relationships between trauma and suicide ideation type, and suicide behavior type. Hierarchical multiple regressions evaluated relationships between trauma and aggression severity, symptoms and functioning. Analyses were performed using SPSS version 25 [54], and significance was determined using *p* < 0.05 due to a priori hypotheses. Significance for analyses comparing trauma history and BPRS factors was determined using the Benjamini–Hochberg procedure with a false discovery rate of 25% to control for multiple comparisons.

## 3. Results

### 3.1. Demographic and Clinical Characteristics of Sample

**Figure 1:**
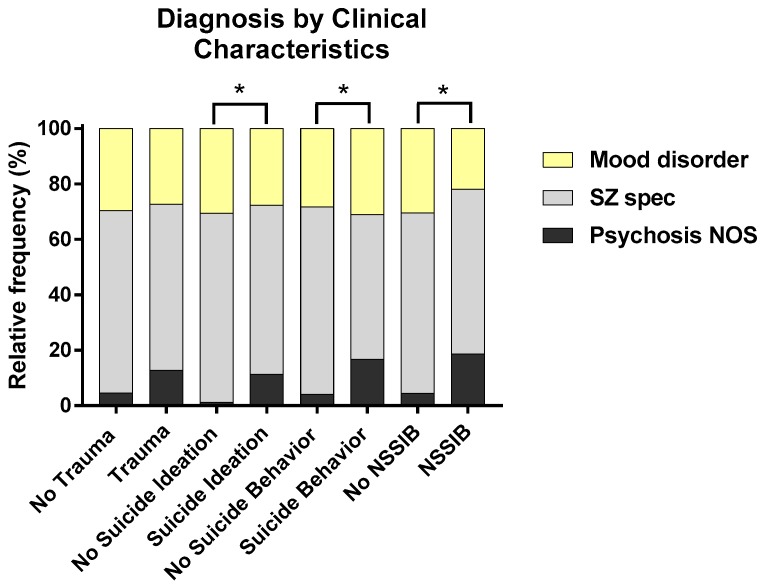
Proportions of participants with and without a history of trauma, suicide ideation, suicide behavior, and non-suicidal self-injury behavior (NSSIB) differed for those with psychosis NOS diagnoses. * *p* < 0.05.

Of the sample (*N* = 187), 29.4% of participants reported a history of trauma on the PCL or during clinical interview. Within this sample, 1.1% reported all three trauma types (emotional abuse, physical abuse, and sexual abuse: *n* = 2), 2.1% reported both physical and sexual abuse (*n* = 4), 3.2% reported both emotional and physical abuse (*n* = 6), 4.3% reported sexual abuse only (*n* = 8), 4.8% reported physical abuse only (*n* = 9), 2.1% reported emotional abuse only (*n* = 4), and 13.4% reported other forms of significant trauma such as exposure to domestic violence, witnessing traumatic deaths or severe medical trauma (*n* = 25). As shown in Table 1, trauma history did not significantly differ by diagnosis, (*X*^2^ = 4.02, *p* = 0.13, *φ_c_* = 0.15), sex, (*X*^2^ = 0.939, *p* = 0.33, *φ* = 0.24), ethnicity (*X*^2^ = 0.02, *p* = 0.88, *φ* = −0.03), race (*X*^2^ = 8.84, *p* = 0.18, *φ_c_* = 0.22), or age (*t* = 0.77, *p* = 0.44, *d* = 0.12). However, there was a trend for history of trauma to differ by diagnosis, with a larger proportion of those with psychosis NOS diagnoses reporting trauma compared to no trauma, as displayed in Figure 1. A similar significant effect was found for suicide ideation, suicide behavior and NSSIB, with a higher proportion of those with psychosis NOS diagnoses reporting these symptoms, as displayed in Figure 1. Additional information on the relationships between participant demographics and clinical characteristics are reported in Supplementary Materials.

### 3.2. Impact of Trauma on Clinical Characteristics

#### 3.2.1. Aggression

**Figure 2:**
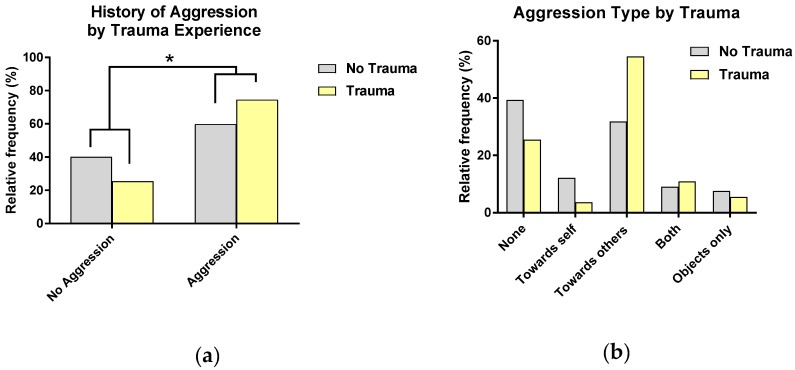
Relationships between trauma history and aggression: (**a**) A higher proportion of those with a history of aggression report having a history of trauma and (**b**) proportions of reported aggression types are similar across trauma no trauma groups, except for aggression towards others where a higher proportion of those reporting aggression towards others reported a history of trauma. * *p* < 0.05.

Of the sample (*N* = 187), 64.2% of participants reported a history of aggression (including verbal and physical aggression towards others and towards self). Additional analyses examining relationships between demographic variables and aggression are presented in the Supplementary Materials. As shown in Figure 2, participants with a history of trauma were 2.05 times more likely to report a history of aggression, and this remained significant when controlling for related demographic variables (ethnicity), (*X*^2^ = 3.923, *p* = 0.05). Depression did not significantly impact the model and so was removed. Further analysis revealed that reported aggression for trauma and no trauma groups differed depending on aggression type (*X*^2^ = 10.63, *p* = 0.03, *φ**_c_*** = 0.03). As shown in Figure 2, those in the trauma group reported more aggression towards others, but not other types of aggression. Trauma history also significantly predicted severity of all types of aggression (*F* = 6.70, *p* = 0.01, *β*= 0.18), and severity of aggression towards objects/others (*F* = 9.85, *p* = 0.01, *β* = 0.23). Depression and sex did not significantly impact either model and so were removed.

#### 3.2.2. Symptom Severity

Those reporting a trauma history had significantly higher BPRS depression symptoms compared to those with no trauma history (*F* = 4.02, *p* = 0.05, *β*= 0.14). No other BPRS factor scores differed between those with and without a trauma history. Additional analyses examining relationships between symptoms and the additional clinical variables are presented in the Supplementary Materials.

#### 3.2.3. Suicide Ideation and Behavior

**Figure 3:**
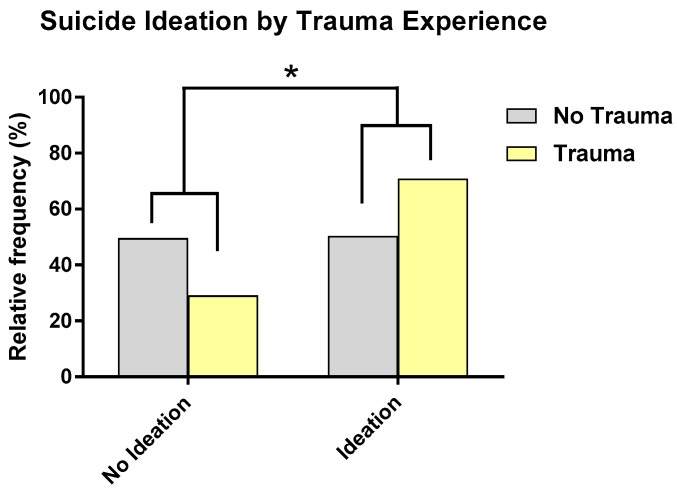
Participants with a history of suicide ideation were more likely to report a history of trauma than those without suicide ideation. * *p* < 0.05.

Of the sample (*N* = 187), 56.1% reported a history of suicide ideation (including 13.9% reporting active suicide ideation with a plan and intent to act) and 22.5% reported a history of suicide behavior (including 13.9% who reported a history of suicide attempts). Additional information on rates of suicide ideation and behavior and analyses examining relationships between demographic variables and suicide ideation and behavior are presented in the Supplementary Materials. As shown in Figure 3, participants with a history of trauma were 2.15 times more likely to report a history of suicide ideation than those without a history of trauma, and this remained significant when controlling for related demographic factors and symptoms (diagnosis and BPRS depression symptoms) (*X*^2^ = 4.49, *p* = 0.03). The impact of trauma history also significantly differed by type of ideation. The odds of those with a trauma history reporting a more severe type of ideation was 1.98 times that of those with no trauma history, a statistically significant effect (*X*^2^ = 5.483, *p* = 0.02).

Suicide behavior was not significantly predicted by trauma history and neither was behavior type. This did not change when controlling for related demographic factors and symptom scores.

#### 3.2.4. NSSIB

**Figure 4:**
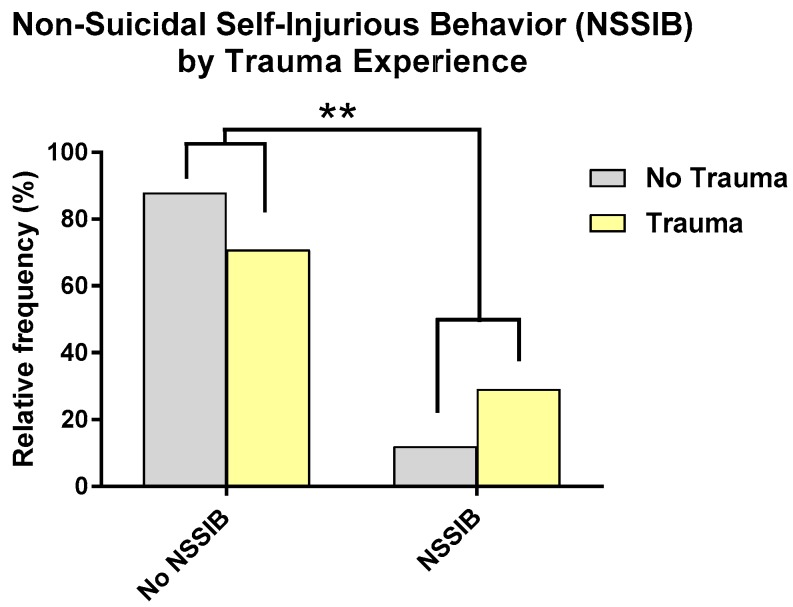
Participants with a history of NSSIB were more likely to report a history of trauma than those without NSSIB. ** *p* < 0.01.

Of the sample of participants (*N* = 187), 17.1% reported a history of NSSIB. Additional analyses examining relationships between demographic variables and NSSIB are presented in the Supplementary Materials. As shown in Figure 4, participants with a history of trauma were 2.45 times more likely to report a history of NSSIB, and this effect remained significant when controlling for related demographic characteristics and symptoms (age and depression) (*X*^2^ = 4.60, *p* = 0.03). Sex and diagnosis did not significantly add to this model, and so were removed. Trauma explained 64% of the variance within the model.

#### 3.2.5. Social and Role Functioning

Of the sample of participants (*N* = 187), 181 had valid functioning scores. Additional analyses comparing demographic variables for social and role functioning are presented in the Supplementary Materials. Baseline social and role functioning scores were not related to trauma history. This did not change when controlling for related demographic factors and symptom scores.

## 4. Discussion

Clinical correlates of psychosis were examined in FEP individuals with and without a trauma history. As hypothesized, history of trauma was associated with increased incidence of aggression, suicide ideation, and NSSIB. It was also associated with increased severity of depression, aggressive behavior, and suicide ideation. Unexpectedly, history of trauma was not associated with a history of suicidal behavior, severity of suicidal behavior, social, or role functioning. These results further support a need for the consideration of trauma within early psychosis assessment and intervention.

### 4.1. Trauma and Symptoms

A history of trauma was associated with higher depressive symptoms, supporting past findings that trauma history is related to depression symptoms in those with early psychosis [27], but contrary to findings that a history of trauma is not related to a Major Depressive Disorder (MDD) diagnosis [55]. It seems plausible that experiencing trauma increases depression symptoms, but not necessarily to the level of clinical MDD. Unexpectedly, a relationship between trauma history and positive psychosis symptoms was not found, which is contrary to other findings that trauma history is related to psychosis symptoms in the general population [55], in CHR individuals [56], and in those experiencing early psychosis [27]. One other study in FEP supports the present finding, with results showing no difference in Scale for the Assessment of Positive Symptoms or Scale for the Assessment of Negative Symptoms scores for FEP with and without a trauma history [19]. This suggests additional factors may complicate the relationship between trauma and positive symptoms, such as recent stressful life events [57]. The present study also found no association between trauma history and negative psychosis symptoms, which is consistent with previous findings that trauma history is not related to negative psychosis symptoms in CHR [56] and that trauma history is not related to Positive and Negative Syndrome Scale negative symptom scores in those with early psychosis [27].

### 4.2. Trauma and Suicide Ideation and Behavior

The finding that FEP individuals with a history of trauma are more likely to report a history of suicide ideation, even when controlling for depression, extends on past findings that a trauma history is associated with suicide ideation in individuals with schizophrenia [17] and CHR individuals [18]. This suggests that a history of trauma may contribute to suicide ideation in FEP, beyond its impact on depression symptoms. Some additional possible mechanisms linking trauma and suicide ideation include maladaptive schemas [57], anxiety [58], or poor social support [58]. However, the present study did not support a relationship between trauma history and suicide behavior, contrary to previous studies in FEP [20]. This difference could be due to methodological differences (such as comparing history of suicide behavior rather than prospective suicide behavior), or cultural differences (past studies were completed on an Australian sample). However, it is important to consider that suicide ideation and behavior may differ in their etiologies. Additional factors worth considering in the etiology of suicide behavior, such as impulsivity or emotion dysregulation [59], were not examined here.

### 4.3. Trauma and Aggression

This study found that FEP participants with a history of trauma were significantly more likely to report a history of aggression than those with no trauma history. This finding is consistent with prior findings that trauma, specifically childhood abuse, is associated with higher prevalence of aggressive behavior in both general and psychosis populations [22,23,60]. The present study also showed that the most prevalent type of aggressive behavior within the trauma group was aggression towards others, and that trauma history is associated with more severe aggression. To our knowledge, this was the first study linking trauma history with severity of aggression in FEP.

Populations with psychosis are more likely to exhibit aggressive behavior in comparison to the general population [61], which may at least be partially due to the high rates of trauma experienced by this population. A history of trauma is associated with increased rates of substance use disorder [62], which is also thought to mediate the relationship between aggression and psychosis [23]. This suggests substance use may be a factor that raises risk for both aggression and psychosis, following the experience of trauma. Additionally, trauma may contribute to aggression by increasing emotional reactivity. Trauma-related cognitions such as the likelihood of being harmed and sense of threat may exacerbate both emotional reactivity and aggression [23].

### 4.4. Trauma and NSSIB

In the present study, a history of NSSIB was associated with a higher likelihood of reporting a history of trauma. This appears to be a new finding. Research on self-harm behavior in psychosis has previously focused on suicidal self-harm. Thus the association between a trauma history and NSSIB in an early psychosis population is uncertain, despite clear evidence NSSIB is increased for those with a trauma history [63]. One possible mechanism linking trauma and NSSIB is impulsivity given childhood trauma is associated with impulsivity as an adult [64], and NSSIB is associated with impulsivity [59]. Another possible explanation is that those who have experienced trauma may have higher emotion reactivity and thus attempt to cope using NSSIB [65].

### 4.5. Trauma and Functioning

In this study, a history of trauma was not associated with higher impairment in social or role functioning compared to those without a trauma history. This supports other studies that found general trauma history was not associated with impaired social or role functioning for those at risk for psychosis [18], and studies in psychosis populations that found an association between trauma history and significantly poorer global assessment of functioning but not specifically social and role functioning [66,67]. This is in contrast to past research on psychosis populations that found a significant difference in functioning across individuals with and without a childhood trauma history [68,69]. Given that our study did not separate childhood trauma and trauma experienced in adulthood, it is possible the impact on functioning is only significant if the trauma occurs during a particular developmental stage [70]. Another possible explanation is that the specific impact of trauma on social and role functioning is less clear for this population, given the already significant impact of psychosis on functioning.

### 4.6. Trauma and Diagnosis

Interestingly, rates of clinical correlates and trauma differed by FEP diagnosis. Specifically, a higher percentage of individuals with a psychosis NOS diagnosis reported a history of trauma, suicide ideation, suicide behavior, and NSSIB compared to individuals with schizophrenia spectrum or mood diagnoses. It seems possible that the presence of these symptoms or behaviors complicates psychosis presentation, making it difficult to fit those individuals into a neat diagnostic category. As such, this population may be more likely to be given a psychosis NOS diagnosis, or Unspecified Schizophrenia Spectrum and Other Psychotic Disorder (based on Diagnostic and Statistical Manual of Mental Disorders 5th Edition) [71]. In addition, given outcomes and trauma exposure vary across disorders, future studies should examine whether the impact of trauma on clinical symptoms varies for different psychotic disorders.

### 4.7. Implications for Theoretical Models of Psychosis

**Figure 5:**
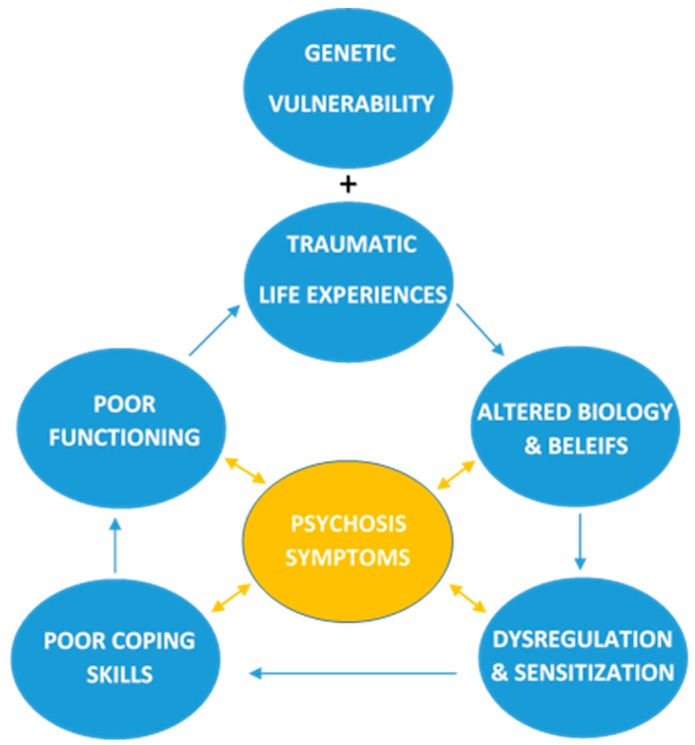
Diagram of how clinical correlates develop and contribute to functioning and psychosis symptoms.

Extending on the model proposed by Mayo et al. [36], we propose (see Figure 5) that the combination of genetic vulnerability and trauma results in an altered developmental trajectory including biological brain changes (e.g., HPA axis sensitization) and changes in beliefs (e.g., schemas that the world is dangerous). These changes result in emotion dysregulation including psychological sensitivity to stress, increased negative affect (e.g., depression), and poor emotion regulation skills. Due to this dysregulation, many people develop maladaptive coping strategies such as suicide ideation, NSSI, and aggression. Use of these coping strategies tends to only be beneficial in the short term, with negative long term consequences such as poor functioning. Poor functioning in turn places people at higher risk for experiencing future trauma and stressful life experiences. Given that each factor following a traumatic life experience in this cascade is thought to be associated with psychosis, it seems likely psychosis symptoms are worsened by a combination of all of these factors. This model highlights trauma-related factors that impact psychosis symptoms, thereby indicating where clinical intervention may be the most effective. For example, improving coping skills may help to improve functioning and prevent future victimization, while also reduce psychosis symptoms.

### 4.8. Implications for Treatment

Given the above model, it appears that in order to reduce suicide ideation, NSSIB, aggression, and other negative emotions such as depression in FEP populations, it is vital to assess trauma history. A thorough assessment of trauma history would allow clinicians to avoid retraumatizing patients, to develop a more comprehensive clinical formulation, and to choose more relevant treatment techniques. In addition, as Figure 5 suggests, there are multiple possible target points for intervention. Present FEP treatment strategies tend to focus on reduction of psychosis symptoms. Our model proposes that treatment for FEP who have experienced trauma may be more effective if treatment targets trauma related factors at an earlier stage. For example, targeting the biological and belief-based changes, or targeting emotion dysregulation and stress sensitivity.

While trauma-focused treatments have been successful in psychosis populations [72,73], these are primarily used to target those with PTSD in the established stage (e.g., more than 5 years) of illness, as opposed to individuals across the early psychosis continuum with a broader definition of trauma history. In addition, these treatments do not specifically target emotion dysregulation resulting from trauma. Treatments such as dialectical behavior therapy (DBT) that directly target emotion dysregulation and associated behaviors (NSSIB and suicidal ideation) could be effective in this population. Finally, there is evidence that targeting specific symptoms (such as depression) in FEP with a history of trauma can improve their functional status [74].

In terms of aggression, it is important to understand the complex mechanisms (such as trauma) that contribute to aggression in psychosis to prevent the stigmatizing belief that psychosis causes aggression. It has been well established that stigma hinders treatment, medication adherence and presentation for treatment [75], which are all associated with higher rates of aggression [76]. Additionally, family-oriented treatment has been demonstrated to be a useful treatment for individuals with psychosis [77], but aggressive behaviors could alienate these support people which are essential for effective treatment of psychosis [78]. Therefore, it is important to recognize when trauma may be contributing to aggressive behaviors and provide appropriate psychoeducation to all parties to ensure engagement in appropriate treatment.

Importantly, while the results support the idea that trauma-focused treatments may reduce symptoms of depression symptoms, suicide ideation, and NSSIB for FEP who have experienced trauma, there is no indication this would also reduce suicide behavior. Current suicide behavior prevention tends to focus on measurement of suicide history and suicide ideation. While suicide ideation is associated with suicide behavior, the majority of those with suicidal ideation do not go on to make a suicide attempt [79]. Thus, attempts to reduce suicide behavior may need to focus on additional factors such as emotion dysregulation [59], and should not rely on measurement of suicidal ideation alone.

### 4.9. Limitations and Strengths

These findings should be considered within the context of methodological limitations. Firstly, history of trauma and aggression were coded retrospectively from clinical charts. As such, this method may have gathered lower rates of traumatic events and aggression than validated questionnaires. Rates of traumatic events were high in the current sample (29.4%), although lower than reports in other FEP samples which range from 34% experiencing traumatic abuse [33] to 75% reporting traumatic life events [27]. However, where possible, specific trauma self-report measures were used to prompt reporting of traumatic events, and clinicians were trained to ask about traumatic events during the clinical interview. The differing rate of traumatic events could also be accounted for by high variability in the measurement and definitions of trauma within studies [18]. Interestingly, rates of aggression reported in the current population (64.2%) were higher than has been reported in other first episode populations (29–36%) [52]. This may indicate that chart review is an effective method of capturing aggressive behavior compared to other techniques such as self-report measurement.

Secondly, trauma, aggression, suicide ideation, and behavior and NSSI were measured retrospectively rather than prospectively. Therefore, it remains unclear if trauma predicts or causes these clinical correlates. The impact of time since exposure to trauma was not examined, which may play a role in the impact of trauma on factors such as functioning [80].

Thirdly, trauma type, repeated trauma exposure, and developmental age when trauma occurred were not included within the current analyses. Thus, we cannot be certain if the listed effects differ by trauma type, or if the effect would differ depending on the age when the trauma occurred and/or how many traumatic events occurred. Past research has found that type of trauma has differing impacts on clinical correlates [20], that the individual’s age at the time of the traumatic event can determine severity of outcomes [70] and that repeated trauma exposure (e.g., complex trauma) can influence likelihood of developing psychosis [81]. Future research should examine these variables in relation to each of the clinical correlates.

Finally, most of the effect sizes reported here are considered small, and this study would be strengthened by additional analyses in larger samples to provide further support for these findings.

### 4.10. Implications for Future Research

Given the importance of reducing rates of depression, suicide ideation, NSSIB and aggression in early psychosis, future research should explore these ideas using a longitudinal design, and more specific self-report measures of trauma history and aggression. Inclusion of possible mediating factors such as emotion dysregulation and stress sensitivity would also be informative for improving targets of trauma-focused treatments. This would allow examination of whether trauma is causing increases in these clinical correlates, and whether the timing of trauma impacts risk.

A greater understanding of the role trauma plays in aggressive behavior in psychosis is essential, as it could lead to more targeted interventions that focus on minimizing aggression by addressing trauma related symptoms. In addition, further research is needed to examine factors that contribute to suicide behavior and functioning, and treatment for these factors should also be incorporated in standard psychosis care.

In conclusion, results show that trauma should be assessed in early psychosis care to support targeted treatment to reduce aggression, depression, suicide ideation, and NSSIB. This could be addressed by including trauma-focused treatments and DBT skills into standard care for FEP with a trauma history. Future longitudinal investigations on possible mediating factors between trauma and other outcomes will support the development of these interventions. Further research is needed in FEP to determine ways to improve functioning and prevent suicide attempts.

## Figures and Tables

**Table 1:** Demographics characteristics of the sample (*N* = 187).

		No Trauma (*n* = 132)	History of Trauma (*n* = 55)
		*N* (%)	
Sex	Male	98 (74.2%)	37 (67.3%)
	Female	34 (25.8%)	18 (32.7%)
Race	Caucasian	87 (65.9%)	31 (56.4%)
	African American	13 (9.8%)	13 (23.6%)
	Asian	15 (11.4%)	5 (9.1%)
	Pacific Islander	4 (3.0%)	0 (0.0%)
	American Indian	1 (0.8%)	0 (0.0%)
	Other	1 (0.8%)	0 (0.0%)
	More than one race	11 (8.3%)	6 (10.9%)
Ethnicity	Hispanic	27 (20.5%)	10 (18.2%)
DSM-IV Diagnosis	Schizophrenia Spectrum	87 (65.9%)	33 (60.0%)
	Mood with Psychotic Features	39 (29.5%)	15 (27.3%)
	Psychotic Disorder NOS *	6 (4.5%)	7 (12.7%)
		Mean (SD)	
Age	Years	19.41 (4.11)	18.91 (3.94)

* Not Otherwise Specified.

## Supplementary Materials

The following are available online at https://www.mdpi.com/2077-0383/8/7/1082/s1.
