# Supplementary material for: A History of Trauma is Associated with Aggression, Depression, Non-Suicidal Self-Injury Behavior, and Suicide Ideation in First-Episode Psychosis
Source: J Clin Med. 2019 Jul 23;8(7):1082. doi: 10.3390/jcm8071082 (PMC6678563; doi:10.3390/jcm8071082)
Supplement: Supplementary file 1 [file jcm-08-01082-s001.pdf]

Supplementary Material for: A history of trauma is associated with aggression, depression, non-suicidal self-injury behavior and suicide ideation in first episode psychosis

Additional information on relationships between demographics and clinical variables

Aggression

Of the sample (N = 187), 64.2% of participants reported a history of aggression (including verbal and physical aggression towards others and towards self). This history of aggression did not differ by diagnosis (X^2 = 3.85, p = 0.15), sex (X^2 = 0.22, p = 0.64), race (X^2 = 5.62, p = 0.47),age (t = 0.69, p = 0.49) or symptoms (p > 0.05). Aggression history did differ by ethnicity, with those identifying as Hispanic significantly more likely to report an aggression history (X^2 = 5.74, p = 0.02).

Suicide Ideation and Behavior

As shown in Table S1, 56.1% of the sample (N = 187) reported a history of suicide ideation (including 13.9% reporting active suicide ideation with a plan and intent to act) and 22.5% reported a history of suicide behavior (including 13.9% who reported a history of suicide attempts). Suicide ideation did not differ for sex (X^2 = 2.00, p = 0.16), ethnicity (X^2 = 0.41, p = 0.52), race (X^2 = 3.47, p = 0.75), or age (t = 1.33, p = 0.19). As shown in Table S1, suicide ideation did differ by diagnosis (X^2 = 7.42, p = 0.02), with a larger proportion of those with psychosis NOS diagnoses reporting ideation compared to no ideation, when compared to those with schizophrenia spectrum or mood disorder with psychotic features diagnoses. Suicide behavior did not differ for sex (X^2 = 0.10, p = 0.75), ethnicity (X^2 = 0.01, p = 0.93), race (X^2 = 2.03, p = 0.92), or age (t = 0.56, p = 0.55). Suicide behavior also differed by diagnosis (X^2 = 8.61, p = 0.01); with a smaller proportion of those with schizophrenia spectrum or mood with psychotic features diagnoses reporting a history of suicide behavior than those with a psychosis NOS diagnosis. Those reporting a history of suicide ideation and behavior had significantly higher BPRS depression symptoms (p < 0.001). Those reporting a history of suicide behavior had significantly higher BPRS positive symptoms, (F = 4.02, p = 0.05), compared to those with no suicide behavior.

Table S1. Most severe types of suicide ideation and behavior reported by the sample.

Type of Suicide Ideation	*N*	% of sample
No ideation	82	43.9%
Wish to be dead	26	13.9%
Non-specific active suicidal thoughts	20	10.7%
Active suicidal ideation with thoughts of method but without intent to act	20	10.7%
Active suicidal ideation with some intent but no plan	13	7.0%
Active suicidal ideation with plan and intent	26	13.9%
Type of Suicide Behavior		
No behavior	147	78.6%
Preparatory behavior	3	1.6%
Aborted attempt	7	3.7%
Interrupted attempt	4	2.1%
Actual attempt	26	13.9%

NSSIB

Of the sample of participants (N = 187), 17.1% reported a history of NSSIB. NSSIB did not differ for sex ($X^2 = 0.48$, $p = 0.48$), ethnicity ($X^2 = 0.16$, $p = 0.69$) or race ($X^2 = 1.31$, $p = 0.97$). NSSIB did differ by age ($t = 2.518$, $p = 0.04$), with younger participants reporting more NSSIB. NSSIB also differed by diagnosis ($X^2 = 6.71$, $p = 0.04$), with a smaller proportion of those with schizophrenia spectrum or mood with psychotic features diagnoses reporting NSSIB than those with a psychosis NOS diagnosis. Those reporting a history of NSSIB had significantly higher BPRS depression symptoms ($p < 0.001$).

Functioning

Of the sample of participants ($N = 187$), 181 had valid functioning scores. Baseline social functioning scores did not differ by diagnosis, ethnicity, or age ($p > 0.05$) but it did differ by sex ($F = 7.94$, $p = 0.01$). Females reported a higher mean social functioning score at initial presentation. Lower social functioning was also related to higher BPRS Positive Symptoms ($r = -0.258$, $p < 0.001$), BPRS Agitation symptoms ($r = 0.165$, $p = 0.03$) and BPRS Negative Symptoms ($r = -0.266$, $p < 0.001$). Baseline role functioning scores did not differ by sex or ethnicity ($p > 0.05$), but did differ by diagnosis, ($F = 4.79$, $p = 0.01$), and age ($r = -0.28$, $p < 0.001$). Older participants had lower role functioning scores, and those with psychosis NOS diagnoses had higher role functioning scores than the other diagnostic groups. Lower role functioning was also related to higher BPRS depression symptoms ($r = 0.254$, $p = 0.01$) and higher BPRS Negative symptoms ($r = -0.256$, $p = 0.01$).
